# Vasculotide, an Angiopoietin-1 mimetic, reduces acute skin ionizing radiation damage in a preclinical mouse model

**DOI:** 10.1186/1471-2407-14-614

**Published:** 2014-08-26

**Authors:** Elina Korpela, Darren Yohan, Lee CL Chin, Anthony Kim, Xiaoyong Huang, Shachar Sade, Paul Van Slyke, Daniel J Dumont, Stanley K Liu

**Affiliations:** Biological Sciences, Sunnybrook Research Institute, Sunnybrook Health Sciences Centre, 2075 Bayview Ave, Toronto, ON M4N 3M5 Canada; Department of Medical Biophysics, University of Toronto, 101 College St, Toronto, M5G 1L7 Canada; Department of Physics, Ryerson University, 350 Victoria St, Toronto, M5B 2K3 Canada; Department of Medical Physics, Odette Cancer Centre, Sunnybrook Health Sciences Centre, 2075 Bayview Ave, Toronto, M4N 3M5 Canada; Department of Radiation Oncology, University of Toronto, 149 College St, Toronto, M5T 1P5 Canada; Department of Pathology, Sunnybrook Health Sciences Centre, 2075 Bayview Ave, Toronto, M4N 3M5 Canada; Department of Laboratory Medicine and Pathobiology, University of Toronto, 1 King’s College Circle, Toronto, M5S 1A8 Canada

**Keywords:** Radiotherapy, Skin, Acute radiation toxicity, Endothelial cells, Tie2, Angiopoietin-1, Inflammation, Diffuse reflectance spectroscopy, Wound healing, Vasculotide

## Abstract

**Background:**

Most cancer patients are treated with radiotherapy, but the treatment can also damage the surrounding normal tissue. Acute skin damage from cancer radiotherapy diminishes patients’ quality of life, yet effective biological interventions for this damage are lacking. Protecting microvascular endothelial cells from irradiation-induced perturbations is emerging as a targeted damage-reduction strategy. Since Angiopoetin-1 signaling through the Tie2 receptor on endothelial cells opposes microvascular perturbations in other disease contexts, we used a preclinical Angiopoietin-1 mimic called Vasculotide to investigate its effect on skin radiation toxicity using a preclinical model.

**Methods:**

Athymic mice were treated intraperitoneally with saline or Vasculotide and their flank skin was irradiated with a single large dose of ionizing radiation. Acute cutaneous damage and wound healing were evaluated by clinical skin grading, histology and immunostaining. Diffuse reflectance optical spectroscopy, myeloperoxidase-dependent bioluminescence imaging of neutrophils and a serum cytokine array were used to assess inflammation. Microvascular endothelial cell response to radiation was tested with *in vitro* clonogenic and Matrigel tubule formation assays. Tumour xenograft growth delay experiments were also performed. Appreciable differences between treatment groups were assessed mainly using parametric and non-parametric statistical tests comparing areas under curves, followed by post-hoc comparisons.

**Results:**

*In vivo*, different schedules of Vasculotide treatment reduced the size of the irradiation-induced wound. Although skin damage scores remained similar on individual days, Vasculotide administered post irradiation resulted in less skin damage overall. Vasculotide alleviated irradiation-induced inflammation in the form of reduced levels of oxygenated hemoglobin, myeloperoxidase bioluminescence and chemokine MIP-2. Surprisingly, Vasculotide-treated animals also had higher microvascular endothelial cell density in wound granulation tissue. *In vitro,* Vasculotide enhanced the survival and function of irradiated endothelial cells.

**Conclusions:**

Vasculotide administration reduces acute skin radiation damage in mice, and may do so by affecting several biological processes. This radiation protection approach may have clinical impact for cancer radiotherapy patients by reducing the severity of their acute skin radiation damage.

**Electronic supplementary material:**

The online version of this article (doi:10.1186/1471-2407-14-614) contains supplementary material, which is available to authorized users.

## Background

Despite technology-driven improvements in cancer radiotherapy (RT) [1], radiation toxicity remains a significant clinical issue that influences treatment outcome, patient quality of life and survivorship. For example, modern RT methods may result in severe acute skin reactions in about 30% and 60% of breast or head and neck cancer patients, respectively [2, 3]. Severe damage such as desquamation, or skin breakdown, can complicate future tissue reconstruction efforts [4] or necessitate treatment interruptions that compromise tumour control or cure [5]. Targeting the biological determinants of radiation damage is an approach to improving these outcomes. However, to date, these side-effects are managed non-specifically by medicated ointments and dressings which do not prevent damage manifestation. Many investigated radioprotective agents are supported by weak clinical evidence at best according to a recent meta-analysis [6]. The only clinically recommended radiation protectant amifostine has shown efficacy in reducing the severity of acute mucositis and chronic xerostomia [7, 8]. However, the delivery logistics of this radiation protectant coupled with its adverse effects cause patients to have low compliance with its use [9]. Therefore, RT side-effects remain a significant issue for patients surviving with and beyond a cancer diagnosis.

Denham & Hauer-Jensen reviewed the continuum of radiotherapeutic wound development [10]. Ionizing radiation (IR) elicits an immediate inflammatory response and epithelial progenitor cell apoptosis that can lead to failure of tissue barrier function and subsequent desquamation. An influx of immune cells contributes to debris clearance and subsequent granulation tissue neovascularisation that replaces the damaged tissue. Re-epithelialization of the wound bed begins and healing takes longer than in non-irradiated tissues [11].

Microvascular perturbations such as apoptosis, inflammatory activation and loss of proliferative capacity, are increasingly described as mediators in the continuum of IR damage development. In the context of irradiated skin, endothelial cell-protecting strategies have also reduced the severity of skin reactions. Holler *et al.* found that pravastatin reduced BALB/c mouse skin damage along with diminished endothelial cell activation, cytokine release and neutrophil recruitment [12]. Although irradiated skin exhibits reduced endothelial angiogenic capacity [13], Maxhimer *et al.* found that preventing loss of endothelial proliferative capacity and reducing apoptosis with an anti-CD47 morpholino also protected skin of C57BL/6 mice from radiation damage [14].

Given that tempering the microvascular response to IR is a targeted approach to normal tissue radiation protection, we were interested in investigating the potential radiation protection by a novel endothelial cell-targeted preclinical compound. Vasculotide (VT) was designed as a four-armed, polyethylene glycol (PEG)-backboned structure, with each arm attached to a Tie2 receptor-binding peptide. Tie2 is a receptor tyrosine kinase that is found almost exclusively on endothelial cells and a subpopulation of hematopoietic stem cells (Tie2 signaling biology reviewed in reference [15]). VT treatment has previously been shown to lengthen survival and prevent endothelial barrier leakage during endotoxemic lung injury [16]. VT also reduced endothelial cell activation and the presence of pro-inflammatory (TNF-α and IL-6), neutrophil-recruiting (KC/CXCL1 and MIP-2/CXCL2) and macrophage-recruiting (MCP-1/CCL2) cytokine levels in serum and peritoneal lavage of septic mice [17]. A structurally modified VT design also enhanced diabetic wound healing [18]. These findings mirror previous characterizations of the Tie2 endogenous ligand Angiopoietin-1 (Ang1), which is context-dependently opposed by Ang2, another Tie2 ligand [19, 20]. Ang1 promotes endothelial cell survival [21, 22], endothelial barrier integrity [23, 24], suppresses inflammation [25, 26], and supports effective tissue-repairing angiogenesis [27–29]. Ang1 variants, such as a pentameric cartilage oligomeric matrix protein (COMP-) Ang1 [30], are often utilized in lieu of the endogenous protein due to Ang1 multimer instability [31].

Few earlier publications have reported that Ang1 variants protect against radiation damage. An Ang1 chimera inhibited endothelial cell apoptosis *in vitro* through phosphatidylinositol-4,5-bisphosphate 3-kinase (PI3K) signaling [32]. COMP-Ang1 prevented gastrointestinal microvascular endothelial cell apoptosis 4 h after total body irradiation and delayed subsequent animal death [33]. Lastly, adenoviral overexpression of COMP-Ang1 in mice exposed to total body irradiation prevented marked bone marrow hypocellularity and apoptosis, thereby preventing IR-induced myelosuppression [34]. Since administration of Ang1 variants counter radiation-induced microvascular perturbations and tissue damage, we hypothesized that VT would protect the microvasculature in the context of skin radiation damage development and reduce normal tissue toxicity.

In the present study, we utilized a preclinical murine model of acute skin IR toxicity to assess the potential radiation protective effect of VT. We investigated the effect of VT on IR-induced inflammation, the subsequent wound healing and *in vitro* endothelial cell survival and function. We also assessed the potential of VT interfering with tumour control by RT.

## Methods

### VT administration

VT’s Tie2-binding peptide sequence HHHRHSF was previously discovered in a phage display array [35]. Peptides were attached by an additional N-terminal cysteine and maleimide to a tetrameric 10 kDa PEG backbone. VT was produced by Bachem (Torrance, CA, USA) and graciously supplied resuspended in phosphate-buffered saline (PBS) by Drs. Paul Van Slyke and Daniel Dumont (Toronto, ON, Canada). 10 μg kg^−1^ VT (200 ng per mouse) or PBS was administered intraperitoneally in 50 μl volumes 24 h and 1.5 h before irradiation and then every other day until the end of the experiments. In the variable VT administration scheduling experiment, mice were administered PBS continuously, given VT 24 h and 1.5 h before irradiation only (“pre VT”), given VT continuously (“continuous VT”) or given VT starting 2 days after 35 Gy irradiation (“post VT”). For the days that VT was not administered, PBS was given instead.

### Animal handling and sacrifice

Animals were handled in accordance with protocols approved by the Sunnybrook Research Institute Animal Care Committee review process. Seven-week old female athymic nude mice (Charles River Canada) were distributed evenly by weight into different treatment groups. Animals were sacrificed by cervical dislocation at various time points. In preparation for irradiation, lead shielding was placed over the animal, loose flank skin was pulled out through an opening in the shielding and gently taped down onto a plexiglass platform outside the shielding. The exposed flank skin was irradiated within a Faxitron (CP160, Faxitron X-Ray Corp., Wheeling, IL, USA) 0.11 m from the 160 kVp x-ray source for 2.5 min with 6.3 mA, delivering 40 Gy to 4 cm^2^ of skin surface area (a total of the top and bottom surfaces of exposed skin). The 35 Gy dose was delivered using the above settings for 2.2 min.

### Skin damage assessment

Radiation skin damage score, desquamated wound size and body weight were evaluated approximately every other day. Radiation skin damage scores were assigned using a murine skin radiation damage grading scale slightly modified from a previously published scale [36, 37]. Desquamated wound area was determined by taking photographs of wounds using a TG-820 Olympus digital camera and outlining wound surface areas using ImageJ (NIH, Bethesda, MD, USA). To determine if the group medians (skin scores) or means (wound areas) differed from each other over all, the area under each individual animals’ plotted skin score and desquamated area was quantified. The VT group median or mean was divided by the PBS group median or mean to get the area under the curve reduction ratio (AUC RR).

### Diffuse reflectance optical spectroscopy (DOS)

Measurements were performed on days 0 (baseline, a few hours before irradiation), 5, 9, 12 and 28. To minimize movement during DOS readings, mice were anaesthetised during measurements with 1.5% isoflurane. The irradiated skin area was probed for 1 to 3 s at five different spots in a similar configuration for each mouse. Readings were performed in the absence of ambient incandescent light. Technical set-up and raw data processing were performed as previously described [38]. Briefly, broadband light is emitted from the probe source into the skin, light is reflected back into the probe sensor, the raw spectrum is processed and then fitted with a curve. Deoxygenated and oxygenated (oxy-) hemoglobin (Hb) reflect light of a certain wavelength giving distinct peaks around 550 – 600 nm. These species determine the values of the saturated hemoglobin (StO_2_) and total Hb parameters. The best fitting parameter value contributions to the raw spectra were determined by an iterative algorithm using MatLab’s Isqcurvefit function. The equation StO_2_ x Hb = oxyHb was used to obtain oxyHb values.

### Myeloperoxidase (MPO) bioluminescence imaging

In one experiment, 35 Gy-irradiated and non-irradiated mice were imaged longitudinally 6 h, 24 h, 48 h, 72 h, 10 days and 13 days after IR. In another experiment, animals were only imaged on day 23 after IR. Neutrophils were detected by oxidized luminol light emission: luminol can be oxidized by reactive oxygen species via MPO catalysis and by MPO’s product hypochlorite. Luminol sodium salt (Sigma-Aldrich, Milwaukee, WI, USA) was reconstituted in Dulbecco’s PBS right before use and was administered as 200 mg kg^−1^ intraperitoneally as previously described [39]. Briefly, animals were anaesthetized with isoflurane and imaged in the Xenogen 100 IVIS Spectrum (Caliper Life Sciences) *in vivo* optical imaging system using the following settings: 60 s exposure time, f/stop 1, medium binning, field of view E and subject height 1.5 cm. Bioluminescent signal from manually placed 4 cm^2^ circular contours of the irradiated areas peaked 7 min after luminol injection. A region corresponding to the location of the irradiated animals’ wounds was also outlined manually on non-irradiated control mice. The mean luminescence of each group was normalized to the irradiated PBS-treated group values.

### Histology and immunohistochemistry

When mice were sacrificed, the irradiated wound areas were excised and fixed for 24 h in 10% formalin at room temperature. Tissues were paraffin-embedded, sectioned into 6 μm-thick slices and stained with haematoxylin and eosin (H&E). Neutrophils were identified by their polymorphonuclear morphology and staining pattern and counted in twenty high power fields (HPF, 400× magnification) per slide, per mouse (four mice per irradiated group, three in the non-irradiated group). Day 14 sample immunostaining for CD31 (Santa Cruz) and CD45 (LCA type, BD Pharmingen^TM^) was performed using the ImmPRESS detection system (Vector Labs) and DAB (DAKO), and counterstained with haematoxylin. Micrographs of CD31+ (100× magnification) and CD45+ (200× magnification) immunostaining were quantified using experimentally derived red, green and blue colour thresholding in ImageJ. The ratios of threshold pixels to total pixels in regions of interest within three (for CD31) or six to seven (for CD45) random sections per slide were averaged. Day 28 wound healing qualitative description of H&E slides was provided by a dermatopathologist at Sunnybrook Health Sciences Centre.

### Serum cytokine array

Blood was collected by cardiac puncture from animals sacrificed on days 2, 5 and 28 after 40 Gy. Blood was clotted at room temperature for 30 min (day 2 and 5 samples) or 2 h (day 28 samples) and centrifuged at 1000 *g* for 15 min at 4°C. Serum was aliquoted and frozen immediately at −80°C. Samples were run against a Milliplex 32-plex panel of mouse cytokine and chemokine detection beads (Millipore, St. Charles, MO, USA) by Eve Technologies Corp. assay services (Calgary, AB, Canada) using the Luminex^TM^ 100 system (Luminex, Austin, TX, USA).

### Cell culture

Human dermal microvascular endothelial cells (HMVECs) immortalized with the human telomerase reverse transcriptase catalytic subunit (*hTERT*) as described by Shao & Guo [40] were graciously received from Dr. Shao. HMVEC^*hTERT*^s were grown in Endothelial Basal Medium EBM-2 (Lonza) supplemented with 10% FBS (Gibco), 1 μg ml^−1^ hydrocortisone (Sigma-Aldrich) and 10 ng ml^−1^ EGF (Sigma-Aldrich), maintained in a 20% O_2_, 5% CO_2_, 37°C humidified chamber and split regularly 1:4. The absence of mycoplasma infection was confirmed using a detection kit (Lonza). Experiments involving Ang1 were carried out using purified recombinant human Ang1 reconstituted in PBS according to the manufacturer’s instructions (R&D Systems).

### Clonogenic survival assays

80% confluent cells were trypsinized and the following number of HMVEC^*hTERT*^s were plated in 6-welled plates: 200 (0 Gy), 400 (2 Gy), 800 (4 Gy), 1600 (6 Gy). 16 h later, cells were starved in serum-free media for 4 h, then stimulated for 3 h, and irradiated using the Faxitron at a distance of 0.33 m x-ray source for 1.1, 2.2, or 3.3 min (for 2, 4, 6 Gy, respectively). Plates were fixed and stained with 25% methanol and 0.5% crystal violet 12 days later and colonies of over 50 cells were counted using a light microscope. Plating efficiency-normalized mean surviving fractions and standard deviations (SDs) were plotted on a semi-log scale from three independent experiments, each with three replicates per condition. Using GraphPad Prism 5.0 (GraphPad Software Inc, CA, USA), the linear quadratic model was fit to the experimental data and the areas under the curves (AUCs) were used for statistical analysis and for survival enhancement ratio (SER) calculations (SER = mean VT or Ang1 AUC / mean PBS AUC).

### Matrigel tubule formation assay

80% confluent HMVEC^*hTERT*^s were starved in serum-free media for 4 h, stimulated for 3 h and then irradiated with 4 Gy. Cells were then passaged 1:3 the following day and 1:2 three days after that. The following day, 10^4^ cells were plated onto 50 μl of undiluted growth factor reduced Matrigel (BD Biosciences) on a 96-welled plate. Cells began to form tubules within the first hour, and tubule networks were captured 6 h after plating using a Motic AE2000 light microscope, Moticam 3.0 camera module and Motic Image Plus 2.0 camera software. Each condition consisted of two or more wells and two to four 200× magnification fields of view were captured per well. Mean total network tubule lengths were quantified using ImageJ software. The experiment was performed three independent times.

### Statistical analyses

All experiments were analyzed for statistical significance in the following way unless otherwise specified. *In vitro* experiments consisted of three independent experiments, each with three replicates. Appreciable differences between means were tested for statistical significance using unpaired two-tailed t-tests. *In vivo* and histological experimental results were also evaluated using the specified t-tests.

To determine if overall skin scores varied by group, each group’s individual animal skin scores’ AUC medians were compared using Mann Whitney (two group comparison) or Kruskal-Wallis (multiple group comparisons) tests. To determine whether overall wound sizes varied by group (plotted as mean ± SD), each group’s individual animal wound sizes’ AUC means were compared using the t-test (two group comparison) or 1-way ANOVA test (multiple group comparisons). Irradiated PBS vs. VT-treated group mean oxyHb levels were also compared in this manner. 1-way ANOVA and Kruskal-Wallis tests were followed by Holm’s method or Dunn’s multiple comparisons test (using α = 0.05), respectively, to reduce the likelihood of false positives. Holm’s method was also utilized when additional pair-wise comparisons were made between groups at specific time points after the overall or main group differences were evaluated. Statistical significance levels P < 0.05, P < 0.01, and P < 0.001 are denoted by *, ** and ***, respectively, when they also meet the α-cutoffs in the case of multiple comparisons. “ns” represents ‘not significant’. Animal weights and the MPO time course were evaluated by t-tests at certain time points (followed by Holm’s method) rather than by comparing overall AUCs. Due to the large intra-group variability of cytokine levels, P-values from t-tests between irradiated PBS vs. VT groups are included without multiple comparison corrections.

### Additional methods

Immunoprecipitations (IPs), western blotting and cancer model experimental methods are described in Additional file 1.

## Results

### Continuous VT treatment reduces acute skin IR toxicity manifestation

**Figure 1:**
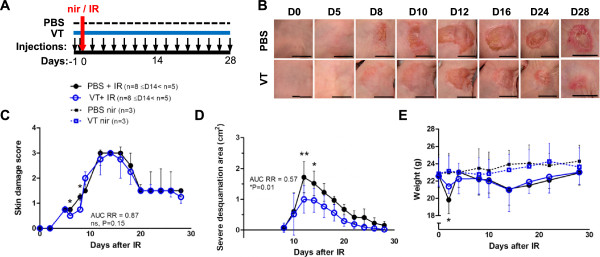
**The effect of VT on irradiation-induced acute cutaneous damage. (A)** Single large 40 Gy fraction of IR and resulting skin toxicity treatment schedule and data collection outline. **(B)** Photographs of radiation dermatitis in PBS (top) or VT-treated (bottom) mice at baseline (day 0) and up to 28 days after irradiation. Scale bars = 1 cm. **(C)** Acute skin damage scores of mice exposed to IR over time expressed as group medians ± interquartile range. **(D)** Absolute surface area of wound moist desquamation and scabbing following irradiation expressed as group means ± SD. **(E)** Mean weights ± SD of PBS/VT-treated irradiated and non-irradiated animals over time. * and ** indicate P < 0.05 and P < 0.01, respectively, and “ns” denotes ‘not significant’.

To investigate the effect of VT on IR-induced acute cutaneous damage, athymic nude mice were treated with PBS or VT intraperitoneally twice before and every other day after a single dose of 40 Gy to the flank skin (Figure 1A). Skin damage development was evaluated using a detailed qualitative acute radiation skin damage scale (Table 1, modified from a previously published scale [36, 37]) similar to grading scales developed for clinical use. 0 represents “normal” and 3.0 signifies “moist desquamation of the irradiated area with possible slight moist exudates”. The wounds reached maximal median damage scores of 3 between days 12 to 16, then healed to score 1.5 (“moist breakdown in one very small area with scaly or crusty appearance”) by day 20, and remained constant until time of sacrifice on day 28 (see reaction progression in both groups in Figure 1B). Although the acute damage scores were similar in both groups on day 9 and onward, there was less erythema on days 6 and 8 in the VT group (damage scores on day 6: PBS 0.75 vs. VT 0.50, *P = 0.017; day 8 PBS 1.37 vs. VT 0.75, *P = 0.032, Figure 1C).

**Table 1:** **Acute radiation mouse flank skin reaction scoring criteria**

Score	Observation
0.00	Normal
0.25	50/50 doubtful if there is any difference from normal
0.50	Very slight reddening
0.75	Definite but slight reddening
1.00	Severe reddening
1.25	Severe reddening with white scale, “papery” aspect of the skin
1.50	Moist breakdown in one very small area with scaly or crusty appearance
1.75	Moist desquamation in more than one small area
2.00	Moist desquamation of larger area: 10% of the irradiated area
2.25	Moist desquamation of larger area: 33% of the irradiated area
2.50	Moist desquamation of larger area: 50% of the irradiated area
2.75	Moist desquamation of larger area: 66% of the irradiated area
3.00	Moist desquamation of most of the irradiated area with possible slight moist exudates
3.25	Moist desquamation of most of the irradiated area with definite moist exudates
3.50	Moist desquamation of the irradiated area with moist exudates, necrosis

Adapted from Douglas & Fowler, 1976; Douglas & Fowler, 2012.

Mean absolute surface areas of severe desquamation (loss of epidermis, ulceration, and subsequent scabbing) in the irradiated VT group were lower overall than in the irradiated PBS group (VT’s AUC RR = 0.57, *P = 0.012). The peak area was also significantly lower on days 12 (PBS 1.72 cm^2^ vs. VT 1.00 cm^2^, **P < 0.010) and 14 (PBS 1.50 cm^2^ vs. VT 0.96 cm^2^, *P = 0.014, Figure 1D) even though the mean irradiated area was the same for both groups. Both irradiated groups had lower body weights compared to their non-irradiated counterparts; however, their weights fully recovered by day 28 (Figure 1E). VT treatment was well tolerated and the irradiated VT group experienced significantly less weight loss 2 days following irradiation compared to the irradiated PBS-treated controls (decrease from baseline body weight by 12.2% for PBS vs. 5.2% for VT, *P = 0.011).

### VT affects local and system inflammatory markers

**Figure 2:**
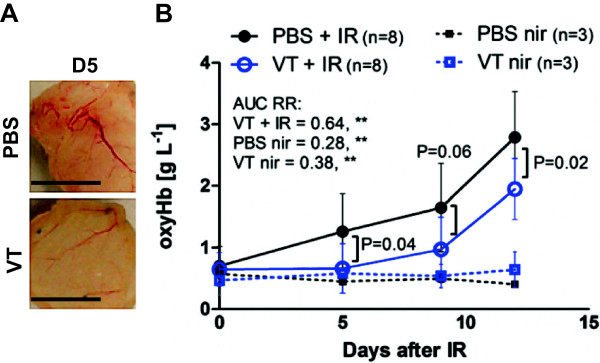
**Imaging time course of local skin erythema after irradiation. (A)** Prominent blood vessel dilation in irradiated PBS-treated, but not in irradiated VT-treated mouse subcutaneous skin photographed on day 5 (scale bars = 1 cm). **(B)** OxyHb levels in irradiated and non-irradiated PBS/VT-treated mice at baseline (day 0) and 5, 9 and 12 days after 40 Gy, expressed as mean ± SD. Expressed as mean ± SD. ** denotes P < 0.01.

Since VT has been shown to have anti-inflammatory effects, we reasoned that VT might reduce IR-induced damage by dampening the inflammatory response. Macroscopically by day 5, the subcutaneous vasculature appeared more inflamed in the irradiated PBS group compared to the VT-treated group (Figure 2A). To quantitatively monitor this inflammation, we performed non-invasive DOS measurements of oxyHb signal in the mouse skin. Vascular oxyHb directly relates to the degree of local erythema and inflammation [41, 42]. Compared to non-irradiated (nir) controls, irradiated PBS-treated mice had increased oxyHb levels on days 5 (PBS + IR 1.26 g L^−1^ vs. PBS nir 0.45 g L^−1^, P = 0.052), 9 (PBS + IR 1.65 g L^−1^ vs. PBS nir 0.50 g L^−1^, *P = 0.024) and 12 (PBS + IR 2.79 g L^−1^ vs. PBS nir 0.41 g L^−1^, ***P < 0.001) after irradiation (Figure 2B). Interestingly, irradiated VT-treated mice trended toward lower oxyHb levels compared to irradiated PBS-treated mice overall (VT’s AUC RR = 0.64, **P = 0.0013) although specific time points did not meet multiple comparison α-cutoffs (day 5: PBS + IR 1.26 g L^−1^ vs. VT + IR 0.66 g L^−1^, P = 0.035; day 9: PBS + IR 1.65 g L^−1^ vs. VT + IR 0.97 g L^−1^, P = 0.06; day 12: PBS + IR 2.79 g L^−1^ vs. VT + IR 1.95 g L^−1^, P = 0.019). This result coupled with the finding that irradiated VT-treated mice had smaller severe wound area formation by day 12 (Figure 1D) suggested that VT decreased the inflammatory burden, thereby reducing the development of a severe wound.

**Figure 3:**
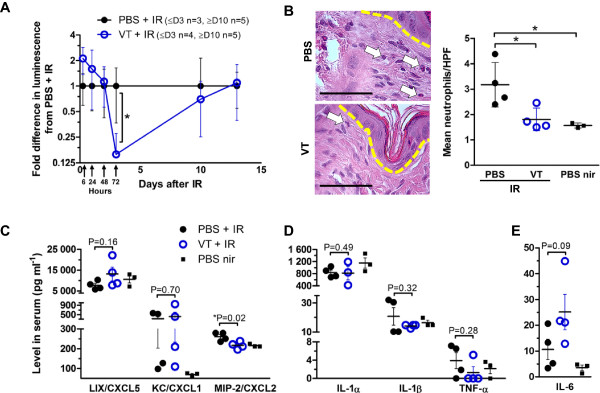
**Early local and systemic inflammatory signs after irradiation. (A)** Irradiated skin MPO detection by bioluminescence in irradiated and PBS/VT-treated mice 6 h, 24 h, 48 h, 72 h, 10 days and 13 days after 35 Gy. Expressed as mean ± SD. **(B)** (Left) Representative H&E-stained skin sections of 40 Gy irradiated and PBS/VT-treated mice on day 5. Dashed line indicates epidermal and dermal boundary. Arrows point out neutrophils under 400x magnification (scale bars = 62.5 μm) and their counts are expressed as the mean of 20 HPFs per mouse ± SD (right). Serum cytokine levels in blood harvested 5 days after 40 Gy skin irradiation: **(C)** neutrophil-recruiting chemokines, **(D)** general pro-inflammatory driver cytokines and **(E)** the pro-inflammatory/neutrophil mobilizing cytokine IL-6, expressed as mean ± SEM. * signifies P < 0.05. Non-significant P-values are also included to aid in evaluation of differences between cytokine levels.

Within two days after an injury, the first immune cells to be recruited to the site of injury are neutrophils and they serve as a hallmark of acute inflammation [43]. However, radiation injury manifests as a complex, prolonged, changing insult, partially because cell death takes place over time [10]. Therefore, we utilized bioluminescence imaging of neutrophil MPO levels to non-invasively and longitudinally quantify neutrophil presence in the skin, as previously applied to irradiated skin by Janko *et al.* [44]. 72 h after irradiation, VT-treated animals exhibited decreased MPO signal (6.4-fold less than the PBS group, *P = 0.043) (Figure 3A). By day 10 they approached the levels of the PBS group (only 1.4-fold less than the PBS group, ns), and by day 13 they were the same. Interestingly, before 48 h, the VT group trended toward greater MPO signal. H&E staining and colorimetric and morphological criteria were used to verify the MPO-based quantification of decreased infiltrated neutrophil levels in mice sacrificed on day 5. There were significantly fewer neutrophils in the irradiated VT-treated group compared to the irradiated PBS-treated group (PBS + IR 3.2 per HPF vs. VT + IR 1.8 per HFP, *P = 0.032) (Figure 3B).

Serum collected from mice sacrificed 2, 5 and 28 days following IR was subjected to a 32-multiplexing cytokine bead array to further elucidate the effect of VT on IR-induced inflammation. Day 2 was chosen as an early time point due to the weight difference seen on day 2. Day 5 was chosen since we saw the earliest difference in oxyHb at that time point, and day 28 was reflective of resolving inflammation due to wound healing.

Since most publications describe neutrophil presence to be detrimental to outcomes following IR exposure [12, 44], we were interested in neutrophil-recruiting chemokine levels (LIX/CXCL5, KC/CXCL1 and MIP-2/CXCL2). On day 5, even before any desquamation had occurred, only MIP-2/CXCL2 levels were decreased by VT following irradiation (*P = 0.02) (Figure 3C). Several previous publications have reported decreased levels of pro-inflammatory driver cytokines IL-1α, IL-1β, TNF-α and IL-6 levels in animals with better outcomes following skin IR exposure [12, 44, 45]. Their levels were not decreased to statistically significant levels by VT treatment by day 5 (Figure 3D).

Among the remainder of the cytokines assayed, several were generally present in very low or below reliably detectable levels (<1 pg ml^−1^: GM-CSF, IFN-γ, IL-3, IL-4, IL-7, IL-12, data not shown). The cytokines within detectable serum levels are grouped according to general function in Additional file 2: Figure S1 based on recent reviews on monocytes [46] and neutrophils [43, 47]. Monocyte-attracting chemokines and pro-inflammatory drivers in the irradiated VT-treated animals trended towards decreased levels overall (across day 2, 5 and 28). Notably, both IL-6 and G-CSF (promoters of neutrophil mobilization from the bone marrow) trended toward increased levels in the VT group across all days.

### VT promotes healing of the IR-induced wound

**Figure 4:**
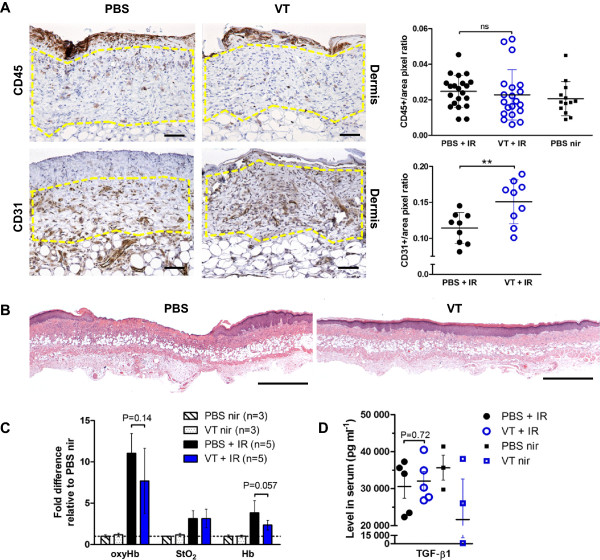
**VT improves IR-induced acute wound healing. (A)** Anti-CD45 (leukocytes, top) and anti-CD31 (endothelial cells, bottom)-stained 200x magnified sections (brown colour) in irradiated PBS and VT-treated animals 14 days after 40 Gy insult. Scale bars = 62.5 μm. Quantified brown pixel to total pixel area (outlined by yellow hatched line) ratios graphed on the right. Expressed as mean ± SD of 3 mice per group. **(B)** H&E-stained histological sections of representative 40 Gy-irradiated and PBS/VT-treated mouse skin 28 days after irradiation. Scale bars = 1 mm. **(C)** Healed 40 Gy-irradiated skin blood parameters (oxyHb, StO_2_, total Hb, mean ± SD) 28 days after IR, normalized to PBS-treated non-irradiated values. **(D)** Profibrogenic cytokine TGF-β levels in blood serum harvested 28 days after 40 Gy irradiation or no irradiation of PBS/VT-treated animals, expressed as mean ± SEM. ** signifies P < 0.01, and “ns” denotes ‘not significant’.

To evaluate the quality of granulation tissue, three mice per irradiated group were sacrificed on day 14 when the degree of qualitative damage peaked. CD45 marks leukocytes of the lymphoid lineage (mainly B cells in this case), granulocytes (such as neutrophils), monocytes and macrophages. There was no significant difference in the amount of CD45+ staining between irradiated PBS and VT treatment group skin sections at this time point (Figure 4A). However, the VT group showed increased CD31+ microvascular endothelial cell staining (VT 0.15 vs. PBS 0.11 staining-to-area ratio, **P < 0.01, Figure 4A).

By day 28, 4 of 5 wounds in the irradiated PBS-treated group remained ulcerated, while only 1 of 5 wounds in the irradiated VT-treated group remained ulcerated (exemplified in Figure 4B with H&E staining). Histological sections from irradiated VT-treated mice also showed better overall healing in terms of a more advanced state of scarring and resolving inflammation (dermatopathologist’s qualitative evaluation). We also determined the hemodynamic characteristics of the healed skin by DOS measurements. OxyHb and Hb readings were lower in the irradiated VT group than in the irradiated PBS group also potentially supporting more resolved inflammation and reduced vascular perfusion, but the differences did not reach statistical significance (Figure 4C). Interestingly, the StO_2_ levels indicated that the wound tissue oxygenation status was the same. This suggested that different oxygen availability was not a contributing factor to the difference in wound healing at this time point. Lastly, high levels of the profibrogenic cytokine TGF-β are associated with poor late toxicity outcomes [48–50], but we did not observe a difference in the profibrotic cytokine levels at the end of the 28-day study (Figure 4D).

### Several VT administration schedules reduce skin radiation damage severity

**Figure 5:**
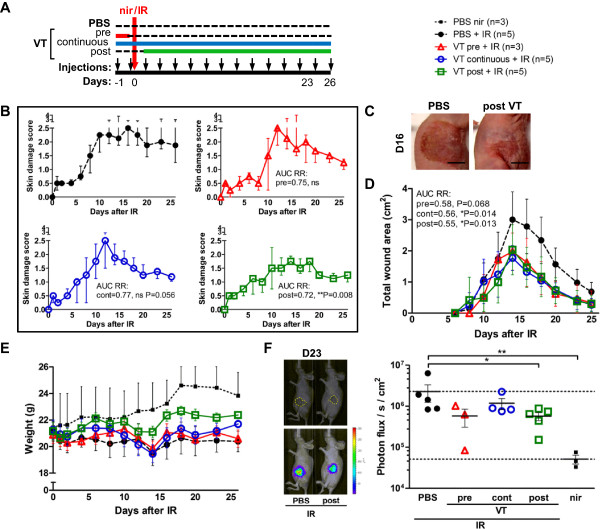
**Time of VT administration relative to irradiation affects the level of damage reached. (A)** Schematic of 35 Gy acute skin toxicity experiment with different VT treatment schedules and data collection outline. **(B)** Acute skin damage scores of mice exposed to IR over time expressed as group medians ± interquartile range. **(C)** Representative photographs of PBS + IR vs. post VT + IR wounds on day 16. Scale bars = 1 cm. **(D)** Absolute surface area of wound involvement following irradiation expressed as group means ± SD. **(E)** Mean weights ± SEM of PBS/VT-treated irradiated and non-irradiated animals over time. Non-irradiated animals are plotted with error bars extending only upwards for a clearer view of the other groups. **(F)** (Left) Photograph of mice with outlined wound and overlay with bioluminescence signal. (Right) MPO bioluminescence detection 23 days after IR and 7 min after luminol injection, expressed as mean ± SEM. * and ** indicate P < 0.05 and P < 0.01, respectively, and “ns” denotes ‘not significant’.

To determine if VT functioned as a direct radioprotector (preventing immediate IR damage) or mitigator (dampening subclinical damage as it develops), we added two experimental groups to the original experimental design (Figure 5A). We also lowered the dose to 35 Gy to improve discrimination/separation of group scores.

The 35 Gy dose resulted in lower median peak damage scores in the PBS + IR group than the 40 Gy dose (2.5 instead of 3). This phenomenon is possible if this dosage range is in the transitional phase (close to the plateau phase) of the sigmoidal probability curve of severe skin reaction development. The post VT + IR group had an overall less severe skin response to the IR than the PBS + IR group (post VT’s AUC RR 0.72, *P = 0.008) and peaked at a median of 1.75 (Figure 5B, pictured in Figure 5C). Additionally, administering VT in two doses before IR (pre VT) reduced the total wound size from the PBS + IR group by the same amount as the continuous and post VT treatments (Figure 5D). However, only the post VT + IR group reached a statistically significant lower peak wound size compared to the PBS + IR group (2.04 cm^2^ vs. 3.01 cm^2^, respectively, *P = 0.022). Pre VT + IR and continuous VT + IR group weights were similar to the irradiated PBS group but the post VT + IR group had the highest weights by day 26 (106% baseline vs. 96.3% of day 0 baseline for post VT + IR and PBS + IR groups, respectively, *P = 0.040) (Figure 5E). As a measure of late inflammation, neutrophil presence was quantified in the skin 23 days after 35 Gy through bioluminescent detection of MPO activity. In comparison to the PBS + IR group, only the post VT group and non-irradiated group had significantly reduced MPO bioluminescence (Figure 5F).

### VT protects endothelial survival and function from IR *in vitro*

**Figure 6:**
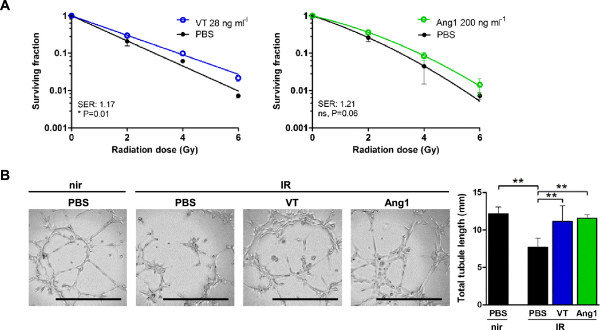
**VT and Ang1 increase survival and function of irradiated immortalized human microvascular endothelial cells. (A)** *In vitro* HMVEC^*hTERT*^ clonogenic survival with 28 ng ml^−1^ VT (left) or 200 ng ml^−1^ Ang1 (right). Expressed as mean ± SD and SERs are indicated. **(B)** Micrographs of HMVEC^*hTERT*^ tubule formation on Matrigel 6 h after plating (left, scale bars = 500 μm). Cells were pre-treated with PBS, 28 ng ml^−1^ VT or 200 ng ml^−1^ Ang1, and 0 or 4 Gy, and results are graphed (right). Expressed as mean ± SD. * represents P < 0.05, ** represents P < 0.01, “ns” represents ‘not significant’.

The increased microvascular endothelial cell density of irradiation-induced wound granulation tissue in VT-treated mice suggested that VT may have improved microvascular endothelial cell survival or function. We determined the effect of VT and Ang1 on irradiated HMVEC^*hTERT*^ clonogenic survival *in vitro*. VT treatment yielded an overall SER for HMVEC^*hTERT*^s of 1.17 (*P = 0.01), or 1.42 (P = 0.06), 1.61 (**P = 0.004) and 3.00 fold (**P = 0.003) at 2, 4 and 6 Gy, respectively (Figure 6A). Ang1 treatment produced a similar SER of 1.21 although it did not reach statistical significance (P = 0.06).

We seeded HMVEC^*hTERT*^s onto growth factor reduced Matrigel to test if VT could improve their angiogenic capacity after irradiation. Non-irradiated PBS-treated cells formed extensive tubule networks reminiscent of capillaries (Figure 6B). Irradiation diminished the total length of the PBS-treated tubule network from 12.1 mm to 7.6 mm (**P < 0.01), but VT or Ang1 treatment allowed extensive networks to form.

### The effect of VT on Tie2 receptor

Although VT has not been reported to have non-specific targets, we sought to confirm that VT activates the intended Tie2 receptor target. By assaying for the pTyr status of immunoprecipitated Tie2, 15 min VT stimulation of HMVEC^*hTERT*^s showed about 2-fold increases of pTyr to total Tie2 signal compared to PBS-stimulated controls (Additional file 3: Figure S2A). Ang1 caused higher increases in HMVEC^*hTERT*^ Tie2 phosphorylation, but both had a stimulatory effect on a downstream PI3K pro-survival pathway (Additional file 3: Figure S2B).

### The effect of VT on tumour response to IR

During cancer RT, a suitable radioprotectant or radiomitigator would reduce radiation-induced damage in normal tissue without protecting tumour cells, thereby improving the therapeutic ratio. To assess whether VT would affect cancer cell survival, we performed *in vitro* clonogenic survival assays and did not detect a survival difference in PBS vs. VT-treated irradiated LS174T and PC3 cells (Additional file 4: Figure S3A,D). As expected, we did not detect protein expression of the VT target receptor Tie2 in these cell lines (data not shown). To evaluate the possibility that VT administration could increase tumour resistance to RT (presumably through protecting the tumour-associated endothelial cells), we conducted tumour xenograft growth delays in athymic mice. Experiments did not yield a difference in *in vivo* xenograft growth kinetics of PBS vs. VT-treated tumour-bearing mice with or without IR, indicating that VT did not interfere with tumour control using IR (Additional file 4: Figure S3B, C, E, F).

## Discussion

Over half of all cancer patients will receive RT, and many of these patients will experience some form of radiation toxicity due to IR absorption by tissues surrounding the tumour target volume. Currently no well-tolerated clinically-approved agents exist to effectively protect against or mitigate radiation toxicity in normal tissues. In our study, we have shown that VT, a previously described Tie2-binding, microvascular endothelial cell-stabilizing compound also improves IR-induced damage outcomes. Using an *in vivo* model of acute radiation skin toxicity, we observed that VT reduced the surface area of IR-induced skin breakdown and affected inflammation and wound healing.

To determine whether VT dampened the early inflammatory wave, we used a quantitative DOS technique to non-invasively and objectively evaluate IR-induced erythema of the skin through oxyHb measurements. DOS has been used to evaluate IR-induced skin erythema in the preclinical [38, 51, 52] and clinical settings [53–55]. Interestingly, compared to PBS treatment, VT reduced the oxyHb parameter in irradiated mice, keeping readings similar to those of the non-irradiated controls during the first 9 days after IR. We propose that this was due to subdued inflammation and vessel dilation rather than a decrease in vessel density given the increased CD31 immunostaining on day 14 in the VT group.

The reduced inflammation is further supported by the dip in MPO bioluminescence starting on day 3 suggesting decreased neutrophil presence in VT-treated animals. It is also supported histologically by signs of decreased inflammation on day 5 in the form of lower absolute neutrophil counts in irradiated VT-treated skin and lower skin reaction scores on days 6 and 8. Additionally, MIP-2/CXCL2, a potent neutrophil chemoattractant released from murine endothelial cells (and immune cells) [56, 57], was slightly reduced by VT treatment in our experiment and in a murine sepsis model treated with VT [17]. Other groups have also demonstrated that decreased neutrophil [12, 44], chemokine [12, 58] and pro-inflammatory cytokine levels [12, 44, 45] are associated with less radiation-induced skin damage. Specifically, antagonizing or inhibiting the receptor of MIP-2/CXCL2 improved survival in a mouse model of radiation-induced alveolitis [59] and sepsis [60].

Consistently increased IL-6 levels in the VT-treated animals seems incoherent with previous reports of skin IR damage, where IL-6 is usually lowered in the group with less damage [12, 45]. IL-6 is secreted by endothelial cells in response to IR [61], can mobilize neutrophils from the bone marrow [62] and enhance neutrophil tissue transendothelial migration by inducing endothelial cell adhesion molecule presentation [63]. Yet early IL-6 signaling may also enable rapid neutrophil tissue clearance [64], which promotes the resolution of acute inflammation [65]. This observation may help explain why MPO bioluminescent imaging detected increased neutrophil presence in VT-treated animals during the earlier time points (6 – 48 h after IR) that diminished soon after. Indeed, a heightened, rapid influx of neutrophils and subsequent less severe skin damage has been reported within a few hours following single large IR doses in mice [66]. IL-6 also has anti-apoptotic activity in endothelial cells against H_2_O_2_-induced stress [67] and IR-induced death [68]. Additionally, IL-6 knockout mice die more readily from total body irradiation [69], mount an exaggerated inflammatory response to skin irritants [70] and exhibit slower wound re-epithelialization than wild-type mice [71]. These anti-apoptotic, pro-survival and pro-wound healing properties may explain why IL-6 levels in the VT-treated irradiated mice corresponded with better acute skin radiation damage outcomes in our experiments.

We found that VT and Ang1 both directly improved clonogenic survival of irradiated HMVEC^*hTERT*^s. Using this radiobiological gold standard assay for cell viability *in vitro*, we demonstrated that endothelial cell reproductive survival following irradiation was enhanced in the long-term with VT pre-treatment. Furthermore, even though endothelial cell angiogenic sprouting capacity is suppressed in the skin by irradiation [13], VT-treatment enabled HMVEC^*hTERT*^s to form more extensive tubule networks *in vitro* on Matrigel. Endothelial cell survival and sprouting conferred by Ang1 are both dependent on AKT signaling [72]; VT and Ang1 stimulation of HMVEC^*hTERT*^s both activated AKT signaling. These *in vitro* findings support the *in vivo* finding that VT-treatment resulted in increased wound vascularity in irradiated mouse skin compared to PBS-treatment.

Other groups have also demonstrated that improvement of irradiated endothelial cell proliferation capacity minimizes soft tissue IR-induced vasculopathies [14, 73]. Better wound healing outcomes of healthy and irradiated tissue have also previously been ascribed to enhanced wound granulation tissue microvascular density [27, 74]. However, increased microvascular density is associated with radiopathology as well; it was increased in the rectal mucosa 6 months after RT completion in prostate cancer patients [75]. It is unknown whether our observed early enhancement in angiogenesis could advance the development of later pathological neoangiogenesis or telangiectasia (characterized by dilated, leaky and fragile blood vessels) since the granulation tissue vascular network subsides as the wound tissue is remodelled. However, since Ang1 and VT have both previously promoted a non-leaky, mature vessel state [18, 30], it would be interesting to determine if VT administration would prevent or treat eventual telangiectasia development.

All treatment administration schedules with VT – either twice before IR, continuously, or starting 48 h post IR – yielded smaller overall wound areas than PBS treatment. The beneficial effects of both pre IR and post IR administration schedules support the interpretation that VT acts through multiple mechanisms: as a radioprotector to reduce the initial damage, and as a radiomitigator by dampening the overall inflammatory response to reduce the damage acquired from the radiation insult.

There are several limitations to consider in the interpretation of our findings. A high IR dose given in a single fraction to murine skin is the standard preclinical radiation skin toxicity model used because it mimics the pathophysiology of severe human cutaneous radiation-induced reactions in an accelerated fashion [76, 77]. However, it may represent a different inflammatory milieu compared to a fractionated schedule, especially when fractionation is overlaid with wound healing [10, 78]. Therefore, the effect of VT on normal tissue damage in our preclinical model may differ in the clinical setting, where fractionation is the standard of RT. Secondly, the athymic mouse lacks a functional T-cell lymphocyte population, resulting in an impaired adaptive immune system. Since we are investigating the early response of skin to irradiation, the response is carried out by leukocytes of the innate immune system (neutrophils, monocytes and macrophages). Reassuringly, the cytokines of most interest to the current study were comparable to levels previously observed in other mouse genetic backgrounds. That said, the often used inbred C57BL/6, C3H and BALB/c mouse strains also polarize the inflammatory response to radiation one way or another (described in reference [79]). The strength of using the nude athymic mouse model for radiation skin damage was its suitability for qualitative assessment of early skin erythema, performing DOS evaluations and human xenograft tumour growth delay experiments. By using the same mouse strain for both normal tissue and tumour radiation response, the intended target vasculature (and the participating immune response) reacted in the context of the same host genetic background.

Although there may be concern that VT will protect the tumour vasculature and hence impair tumour control by RT [80], we did not observe this outcome. The differential effect of VT may be due to differences in properties of endothelial cells residing in normal vs. tumour tissues [81] or the lower radiation dose delivered [82]. It is interesting that we did not observe an effect by VT on tumour growth as Ang1 has previously been reported to suppress tumour growth in some models [83–86]. In the case of tumours (such as gliomas) with Tie2-expressing parenchymal cancer cells, exogenous Ang1 treatment has been reported to aggravate cancer cell invasion [87]. Therefore, VT administration may not be warranted for Tie2-expressing tumour types and its effects should be investigated further in different tumour models.

## Conclusions

**Figure 7:**
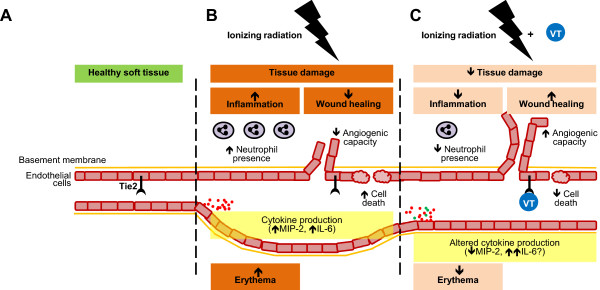
**VT-dependent skin IR protection is associated with decreased inflammatory and enhanced wound healing processes. (A)** In healthy quiescent tissues, Tie2 is found mainly on the microvascular endothelial cell membrane. **(B)** Following irradiation, neutrophil recruitment and cytokine production are evoked and erythema develops. Subclinical tissue damage builds and manifests as skin desquamation. When the broken skin attempts to repair, the wound’s granulation tissue angiogenesis may be impaired during wound healing. **(C)** Continuous VT treatment’s net effect on inflammation on the level of hemodynamics and neutrophil recruitment is a dampening one (lower degree of erythema and neutrophil presence), although how this is achieved beyond decreased MIP-2 levels on the grander scale of the cytokine network (taking into account elevated IL-6, for example) is uncertain. It is also unclear whether the dampened inflammation is from a radioprotective, radiomitigative, or a combinational effect. VT may render radioprotection partially through endothelial cell protection in irradiated skin, but the benefits may be delayed, manifesting only at the time of wound healing through increased clonogenic viability and angiogenic capacity, rather than during subclinical damage. Ultimately, VT treatment reduces the severity of normal tissue radiation damage.

Our research highlights the VT compound with functional similarity to Ang1 as a novel and innovative approach for reducing acute skin radiation damage during RT. Preclinically, VT minimized skin toxicity, reduced associated inflammation and improved wound healing. VT also promoted endothelial cell survival and function. A summary model is illustrated in Figure 7. We envision the utility of VT as a therapeutic agent for clinical sites such as head and neck, lower gastrointestinal, anal and breast cancers. At these locations, acute skin toxicity is of great concern [2, 88, 89]. Our preclinical research suggests that VT may be valuable in these clinical scenarios by providing acute radiation protection to the skin without altering the tumour radiation response. It may be especially useful in patients with increased risk of radiotoxicity from pre-existing conditions of compromised microvascular function such as diabetes or obesity [90, 91].

Acute skin toxicity does not necessarily translate into late skin toxicity. However, when radiation introduces a severe or persistent disruption of tissue that serves a protective or barrier function (i.e. skin or mucosal lining), it may promote secondary trauma to the underlying tissue, and result in the development of consequential late effects [92]. Thus, although not investigated here, it is conceivable that by minimizing acute radiation epithelial barrier disruption, VT treatment may also minimize the development of certain consequential late toxicities. To broaden the translational therapeutic potential of VT, it would be interesting to pursue other specific applications (rectal, bowel or mouth mucosal lining tissue acute toxicities and consequential late effects) in other preclinical models. It would also be interesting to investigate if and how radiation dose delivery (i.e. clinically relevant fractionation) influences VT effectiveness. In the future, we plan to investigate the potential of VT in mitigating late toxicities. These are the major burden of long-term suffering from toxicity for cancer survivors treated with RT, and of greatest concern for both patient and physician.

## Electronic supplementary material

Additional file 1: **Supplemental methods.** (DOCX 17 KB)

Additional file 2: Figure S1: VT alters IR-induced cytokine levels. Cytokine levels in serum harvested from mice 2, 5 and 28 days after 40 Gy cutaneous irradiation. Results are expressed as mean ± SEM in the VT + IR-treated mouse group normalized to PBS + IR-treated mouse group mean levels. “nd” signifies ‘not determined’ (<1 pg ml^−1^) and “nm” signifies ‘not measured’. P-values below 0.2 are indicated for better assessment of differences between samples with great variability and small sample size (day 2 PBS + IR n = 4, VT + IR n = 3; day 5 PBS + IR n = 4, VT + IR n = 4; day 28 PBS + IR n = 5, VT + IR n = 5). (PDF 304 KB)

Additional file 3: Figure S2: VT and Ang1 both activate the Tie2 receptor. **(A)** pTyr and total Tie2 (totTie2) levels were quantified by IP and western blotting. Serum-starved HMVEC^*hTERT*^s stimulated for 15 min with VT or Ang1, and pTyr/totTie2 relative intensities are plotted normalized to PBS. Representative results from 1 of 3 independent experiments. **(B)** Tie2 downstream AKT survival pathway activation by 15 min stimulation by VT or Ang1. Representative results from 1 of 2 independent experiments. (PDF 308 KB)

Additional file 4: Figure S3: Irradiated cancer cell survival and tumour xenograft growth are not affected by VT administration. **(A)** *In vitro* clonogenic survival of LS174T cells with 28 ng ml^−1^ VT treatment expressed as mean ± SD and SER. **(B)** Growth curves of subcutaneous hind limb tumour xenograft following PBS/VT treatment with or without 5 Gy irradiation expressed as mean ± SEM. **(C)** Growth time to reach a 3-fold volume increase from day 1 as mean ± SD. “ns” signifies ‘not significant’. Repeat of assays using PC3 cells for **(D)** clonogenic survival, **(E)** tumour xenograft growth curves with or without 3 x 2 Gy irradiation and **(F)** overall growth time. (PDF 244 KB)

## Abbreviations

Ang1: Angiopoietin-1
AUC RR: Area under curve reduction ratio
COMP: Cartilage oligomeric protein
DOS: Diffuse reflectance optical spectroscopy
Hb: Haemoglobin
HPF: High power field
HMVEC: Human dermal microvascular endothelial cell
IP: Immunoprecipitation
IR: Ionizing radiation
MPO: Myeloperoxidase
nir: Non-irradiated
oxyHb: Oxygenated haemoglobin
PBS: Phosphate buffered saline
PI3K: Phosphatidylinositol-4,5-bisphosphate 3-kinase
pTyr: Phosphorylated tyrosine
PEG: Polyethylene glycol
RT: Radiation therapy/radiotherapy
StO_2_: Saturated haemoglobin
SD: Standard deviation
SEM: Standard error of the mean
SER: Survival enhancement ratio
VT: Vasculotide.
