# Supplementary material for: Vasculotide, an Angiopoietin-1 mimetic, reduces acute skin ionizing radiation damage in a preclinical mouse model
Source: BMC Cancer. 2014 Aug 26;14:614. doi: 10.1186/1471-2407-14-614 (PMC4159535; doi:10.1186/1471-2407-14-614)
Supplement: Supplementary file 1 — Additional file 1: Supplemental methods. (DOCX 17 KB) [file 12885_2014_4810_MOESM1_ESM.docx]

# Additional file 1 – Supplemental methods

## IP and western blotting

Cells were seeded in 10 cm tissue culture plates in 5% FBS-containing media. Once they reached 80% confluence, cells were serum-starved for 90 min in 0% FBS-containing media. Stimulations were done for 15 min with specified concentrations of Ang1 or VT. Cells were scraped and lysed using RIPA lysis buffer (50 mM Tris HCl pH 7.4, 150 mM NaCl, 1% NP-40, 0.1% SDS, 0.5% Na deoxycholate, 100 mM NaF, 1 mM Na_3_VO_4_, and 1 phophostop tablet (Roche) per 10 ml buffer) similar to the protocol described by Bogdanovic *et al* [[1](#_ENREF_1)]. Briefly, samples were left on ice for 10 min, sonicated for 5 min, and then spun in a microcentrifuge at 4ºC at 15 000 rpm for 15 min. 2 μg of Protein A Sepharose^TM^ CL-4B beads (GE Healthcare) pre-coupled to 2.4 ug Tie2 antibody (C-20, Santa Cruz) was used for a 2 h pull-down. Samples were subject to SDS-PAGE gel electrophoresis, transferred onto PVDF membrane and blotted for total Tie2 (33-1, BD Pharmingen) or phosphorylated tyrosine (pTyr, 4G10) (Millipore). Relative pTyr to total Tie2 ratios were determined by densitometry using ImageJ software. The experiment was performed 3 independent times. Total cell lysates were blotted for pAKT (Ser473), total AKT (both from Cell Signaling Technology) and β–actin (Santa Cruz) in two biologically independent experiments.

## Human LS174T and PC3 clonogenic and tumour xenograft growth delay experiments

Human colorectal adenocarcinoma LS174T cells (ATCC, VA, USA) and human prostate cancer PC3 cells (ATCC, VA, USA) were maintained in DMEM (Gibco) supplemented with 10% FBS, 10 U ml^-1^ penicillin and 10 μg ml^-1^ streptomycin (Wisent) in the same chamber conditions as the HMVEC*^hTERT^*s. They were treated identically to HMVEC*^hTERT^*s in preparation for the clonogenic assay with the exception that different amounts of cells were plated (for 0, 2, 4 and 6 Gy: 300, 1200, 4000 and 8000 LS174T cells, respectively; 100, 200, 1000 and 3000 PC3 cells, respectively). For the xenograft experiment, seven-week old female athymic nude mice (Charles River Canada) were injected with human cancer cells (6 x 10^6^ LS174T or 5 x 10^6^ PC3) in 100 μl DMEM to the hind limb. Once tumours reached ~50 mm^3^, mice were treated every other day with 10-12.5 μg kg^-1^ VT (200-250 ng per mouse) or PBS via intraperitoneal injections until they reached ~100 mm^3^. Tumours were then irradiated with a single 5 Gy fraction (LS174T) or 2 Gy fractions on three consecutive days (PC3). Xenograft volumes were determined every few days by caliper measurements and the elliptoid volume equation: (L x W^2^) / 2. Mice were sacrificed when tumours reached three times their initial ~100 mm^3^ volume. Growth kinetics are plotted as mean ± standard error of the mean (SEM), and growth delays are plotted as mean ± SD. Tumour xenograft growth delays were evaluated by t-tests at certain time points.

# Additional References

1. Bogdanovic E, Nguyen VP, Dumont DJ: **Activation of Tie2 by angiopoietin-1 and angiopoietin-2 results in their release and receptor internalization**. *J Cell Sci* 2006, **119**(Pt 17):3551-3560
