# Supplementary figures and images for: Vasculotide, an Angiopoietin-1 mimetic, reduces acute skin ionizing radiation damage in a preclinical mouse model
Source: BMC Cancer. 2014 Aug 26;14:614. doi: 10.1186/1471-2407-14-614 (PMC4159535; doi:10.1186/1471-2407-14-614)

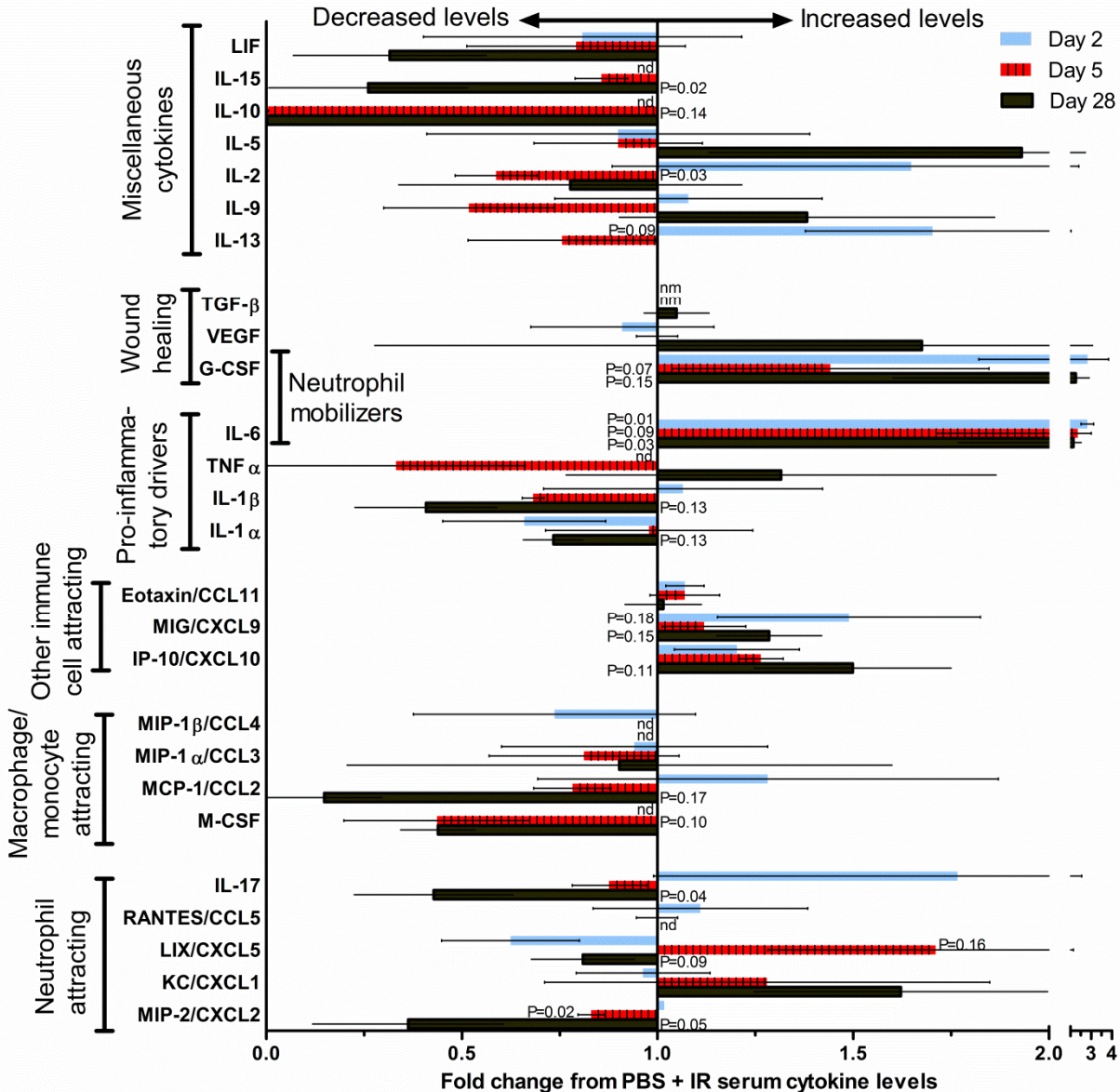

Supplement: Supplementary file 2 — Additional file 2: Figure S1: VT alters IR-induced cytokine levels. Cytokine levels in serum harvested from mice 2, 5 and 28 days after 40 Gy cutaneous irradiation. Results are expressed as mean ± SEM in the VT + IR-treated mouse group normalized to PBS + IR-treated mouse group mean levels. “nd” signifies ‘not determined’ (<1 pg ml−1) and “nm” signifies ‘not measured’. P-values below 0.2 are indicated for better assessment of differences between samples with great variability and small sample size (day 2 PBS + IR n = 4, VT + IR n = 3; day 5 PBS + IR n = 4, VT + IR n = 4; day 28 PBS + IR n = 5, VT + IR n = 5). (PDF 304 KB) [file 12885_2014_4810_MOESM2_ESM.pdf]

**A**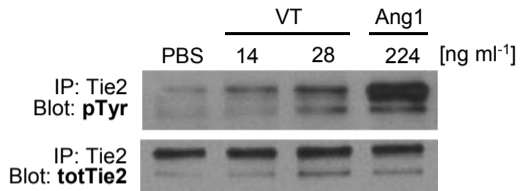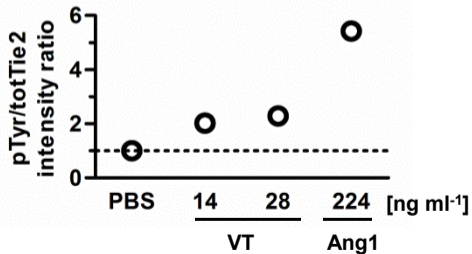**B**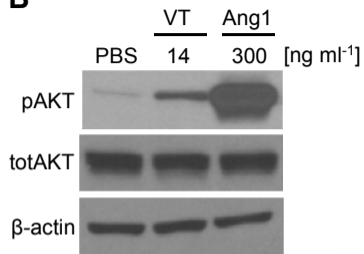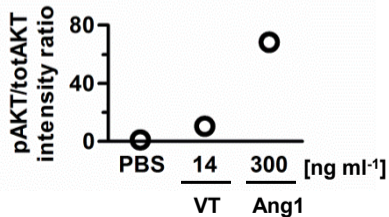

Supplement: Supplementary file 3 — Additional file 3: Figure S2: VT and Ang1 both activate the Tie2 receptor. (A) pTyr and total Tie2 (totTie2) levels were quantified by IP and western blotting. Serum-starved HMVEChTERTs stimulated for 15 min with VT or Ang1, and pTyr/totTie2 relative intensities are plotted normalized to PBS. Representative results from 1 of 3 independent experiments. (B) Tie2 downstream AKT survival pathway activation by 15 min stimulation by VT or Ang1. Representative results from 1 of 2 independent experiments. (PDF 308 KB) [file 12885_2014_4810_MOESM3_ESM.pdf]

**A**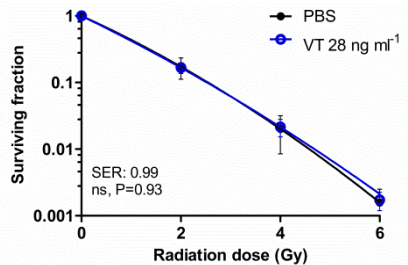**B**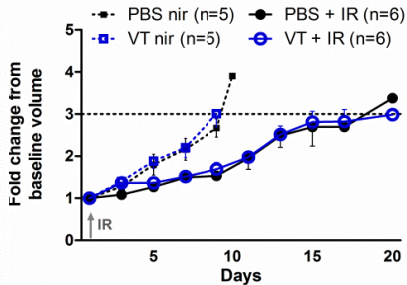**C**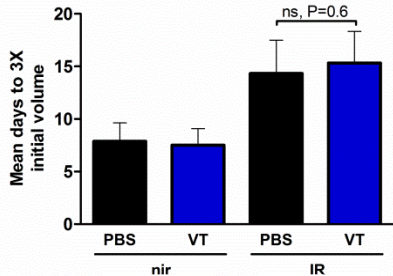**D**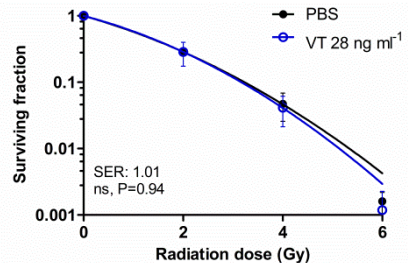**E**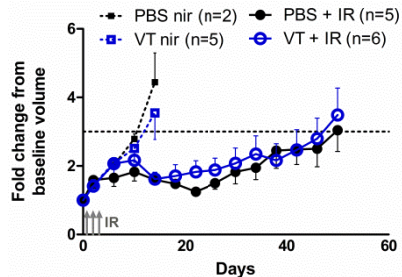**F**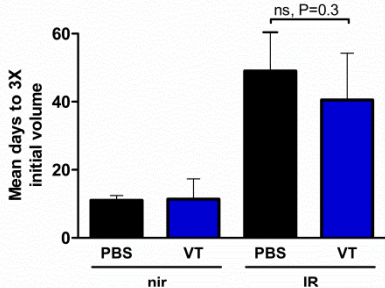

Supplement: Supplementary file 4 — Additional file 4: Figure S3: Irradiated cancer cell survival and tumour xenograft growth are not affected by VT administration. (A) In vitro clonogenic survival of LS174T cells with 28 ng ml−1 VT treatment expressed as mean ± SD and SER. (B) Growth curves of subcutaneous hind limb tumour xenograft following PBS/VT treatment with or without 5 Gy irradiation expressed as mean ± SEM. (C) Growth time to reach a 3-fold volume increase from day 1 as mean ± SD. “ns” signifies ‘not significant’. Repeat of assays using PC3 cells for (D) clonogenic survival, (E) tumour xenograft growth curves with or without 3 x 2 Gy irradiation and (F) overall growth time. (PDF 244 KB) [file 12885_2014_4810_MOESM4_ESM.pdf]
